# Forearm Giant Osteochondromas in a Young Patient With Multiple Hereditary Exostoses: A Case Report

**DOI:** 10.7759/cureus.77295

**Published:** 2025-01-11

**Authors:** Sarmad R Sulaiman, Hossam M Ismail, Shadha ‎ A Al-Zubaidi, Osama F Almaghthawi, Ahmed Alrehaili, Rayan AlArabi

**Affiliations:** 1 Orthopedic Oncology, King Salman Medical City, Medina, SAU; 2 Orthopedics, King Salman Medical City, Maternity and Children Hospital, Medina, SAU; 3 Diagnostic Imaging, Madina Cardiac Centre, Medina, SAU

**Keywords:** elbow, hereditary exostoses, large osteochondroma, proximal radial resection, radial head dislocation

## Abstract

Multiple hereditary exostoses (MHE) is a rare skeletal disorder inherited as an autosomal dominant disorder. It is characterized by widespread multiple osteochondromas that grow near bone growth plates, leading to pain and deformities that significantly impact physical and emotional well-being and disrupt daily activities, social interactions, and psychological health, leading to considerable disability. This case report describes a 15-year-old boy with a family history of MHE who developed a large osteochondroma at his right elbow. We aim to present the surgical management of extraordinarily large-size proximal radius osteochondroma, fortunately, caused by a benign underlying condition despite typically carrying more chances of transformation into malignancy.‎ To the best of our knowledge, it would be the largest proximal radius osteochondroma documented in the literature.

## Introduction

Multiple hereditary exostoses (MHE), also known as hereditary multiple exostoses (HME), is a dominant, autosomal hereditary multifocal rare condition most commonly associated with mutations in two genes, exostosis-1 (EXT1) and exostosis-2 (EXT2), identified in 70-94% of cases, with a prevalence rate of 1 in 50,000 [1-4]. Moreover, there is no sex difference, but males with EXT1 may experience a more noticeable disease. MHE usually appears and is diagnosed in childhood and is characterized by multiple benign osteochondromas with broad (sessile) or pedunculated bases in continuity with the bone marrow of the affected bones, covered by a thin cartilage cap [1-3]. Osteochondromas occur equally in all bones developed from hyaline cartilage; however, around the knee and humerus are the most frequent locations, leading to growth deformities and short stature in many patients. MHE presents a wide range of clinical manifestations. Osteochondromas often occur near growth plates, causing asymmetrical bone elongation, bowing, and shortening, which results in bone deformities, short stature, and restricted joint motion, often accompanied by pain and functional impairment [1-5].

Forearm deformities are common in patients with MHE, occurring in up to 60% of patients. Among these, 20-30% involve subluxation or dislocation of the radial head. Typical deformities include bowing of the radius, relative shortening of the ulna with distortion of the radioulnar joint, fixed ulnar deviation of the wrist, and radial head dislocation. These abnormalities often lead to growth asymmetry, functional limitations, and cosmetic concerns [1-4]. Masada et al. classify the forearm deformity caused by MHE into three groups [5]. Group I involves ulnar shortening and radial bowing due to osteochondromas of the distal ulna. In contrast, Group II includes radial head dislocation, subdivided into Group IIa, a rare type caused by osteochondromas of the proximal radius, and Group IIb, resulting from more distal involvement. Group III is characterized by radial shortening due to osteochondromas of the distal radius [5]. This case report discusses a rare MHE lesion categorized as Group IIa. The most dangerous complication is cancerous transformation, frequently occurring within the cartilage cap, leading to secondary chondrosarcoma in 10% of MHE [4,6]. Infrequently, transformation into osteosarcoma can occur [4,6]. Fortunately, most of these transformations are low to intermediate grade [4,6]. Consequently, the new onset of pain and concerning imaging features, like an increase in the cartilage cap thickness greater than 3 cm in children or 2 cm in adults, might signal malignant transformation [4,6]. This case report highlights the surgical management of rare, large osteochondromas arising from the radial head and distal ulna. The main objectives were to excise the lesion, restore the joint’s range of motion, and prevent potential complications such as pathological fractures, neurovascular injury, or malignant transformation. To the best of our knowledge, this represents the largest reported osteochondromas originating from the forearm bones, offering valuable insights into their clinical presentation, surgical challenges, and treatment outcomes.

## Case presentation

**Figure 1:**
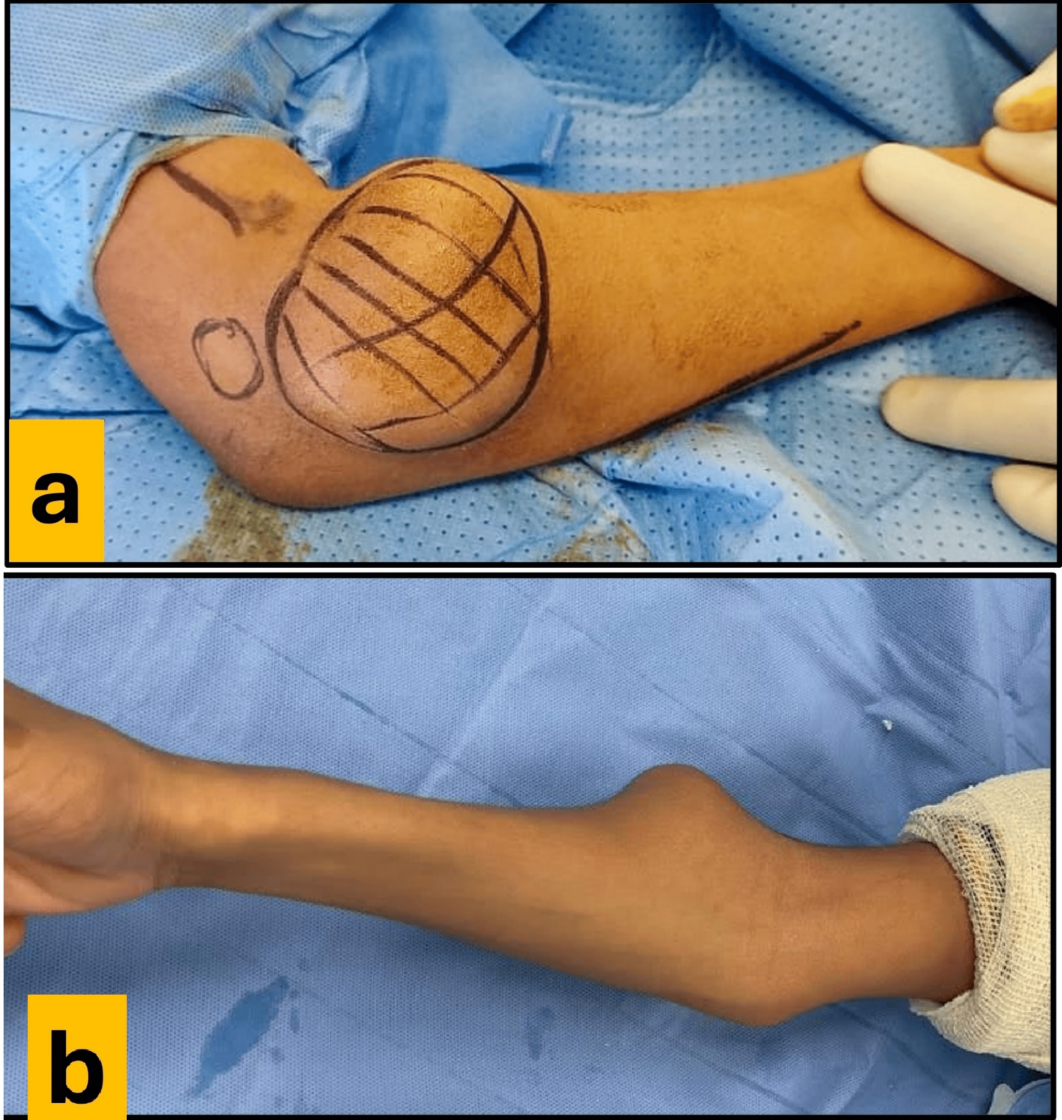
Tumor size. (a and b): Indicating the site and the size of the tumor.

A 15-year-old boy with a family history of MHE presented with pain, deformity, swelling, and limited mobility in the right elbow and the forearm. The symptoms progressively worsened. There was no history of trauma, fever, or joint effusions. The physical examination revealed a nontender, bony, hard mass on the radial side of the right elbow, which was not associated with signs of local inflammation (Figure 1a and Figure 1b)

Furthermore, the right elbow joint and forearm range of motion were significantly limited (elbow 100° flexion, 40° extension, and the forearm was fixed in 20° pronation). Additionally, there is no evidence of the extremity nerves or blood vessel injuries.

**Figure 2:**
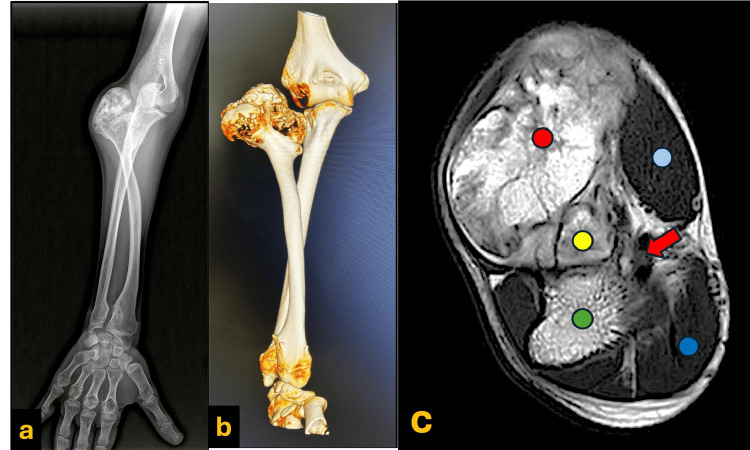
Preoperative plain radiograph, three-dimensional CT reformatted image, and MRI scan. a. Preoperative plain radiograph of the right forearm showed an expansile bony lesion in the proximal end of the right radius with a dislocation of the radio-humeral joint and an increase in the bowing of the radial shaft. Additionally, there is evidence of distal radioulnar abnormal articulation as part of MHE. b. The preoperative three-dimensional CT reformat image showed an expansile bony lesion in the proximal end of the right radius with a dislocation of the radio-humeral joint and bone absorption in the lateral aspect of the humeral trochlea due to the abutment of the bony lesion. The ulna had no significant shortening or angular, varus, or valgus deformities. c. Preoperative MRI demonstrates a cauliflower-like bony outgrowth (Red circle) arising from the lateral metaphysis of the proximal radius (yellow circle), which measures approximately 51x41x48 mm. It shows preserved cortical and medullary continuity with the parent bone. The cartilaginous cap thickness measures about 3 mm. (Green circle= proximal ulna) (Light blue circle= forearm flexors muscles) (Dark blue circle= mobile wad muscles) (Red arrow= neurovascular bundle).

The plain radiographs showed a large, calcified, and heterogeneous irregular mass originating from the radial proximal end and associated with a dislocation of the radio-humeral joint and an increase in the bowing of the radial shaft. Additionally, there is evidence of distal radioulnar abnormal articulation as part of MHE (Figure 2a). The computed tomography (CT) scan showed bone absorption of the lateral aspect of the humeral trochlea due to the abutment of the bony lesion. The ulna had no significant shortening or angular, varus, or valgus deformities (Figure 2b). Moreover, magnetic resonance imaging (MRI) demonstrates a cauliflower-like bony outgrowth arising from the lateral metaphysis of the proximal radius, which measures approximately 51x41x48 mm. It shows preserved cortical and medullary continuity with the parent bone. The cartilaginous cap thickness measures about 3 mm (Figure 2c).

The surgical intervention was considered necessary based on the tumor size, site, and restricted joint mobility. Therefore, the decision was to resect the radial head and the segments occupied by the osteochondroma.

**Figure 3:**
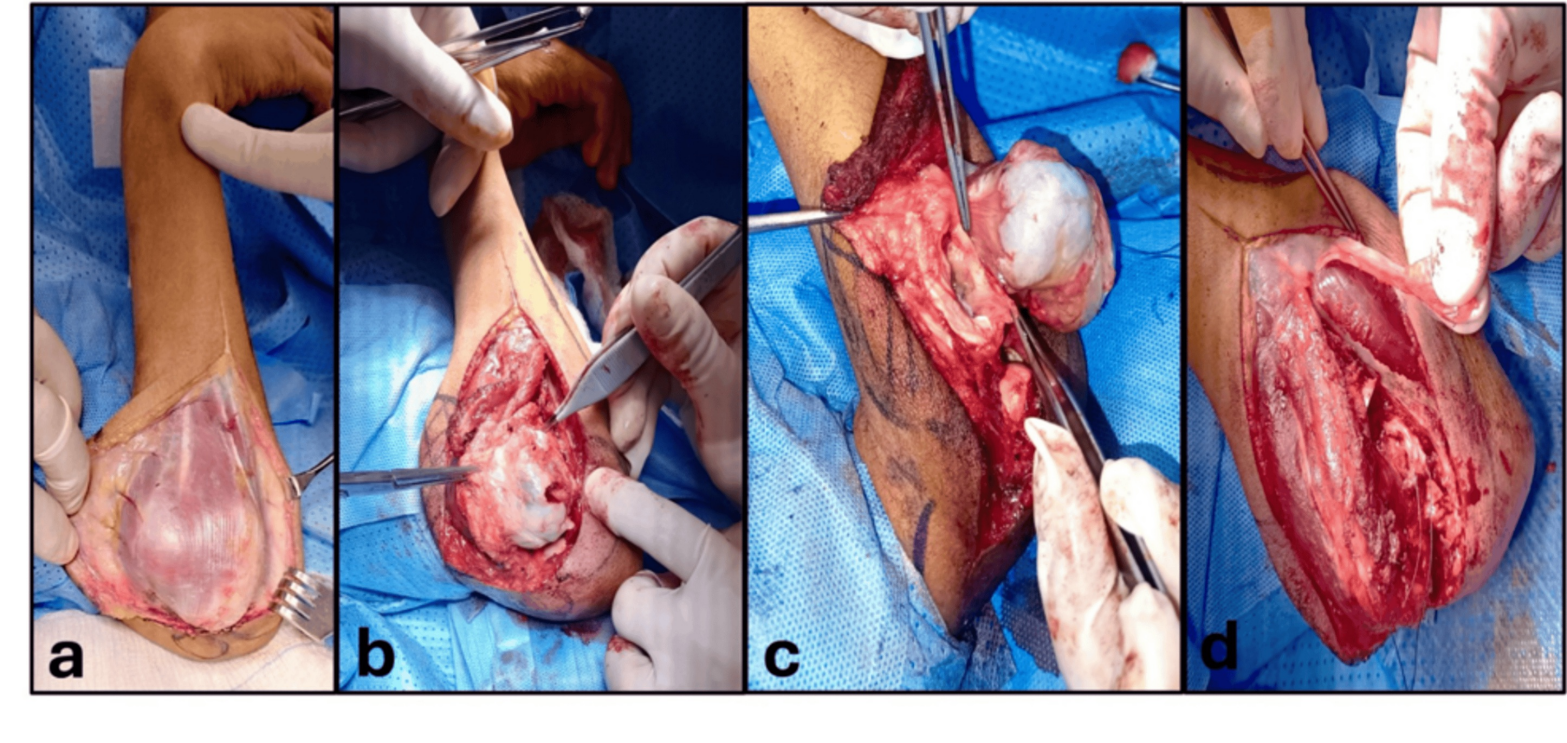
Intraoperative Steps: Skin approach, tumor exposure, posterior interosseous nerve identification, and fascial strip application. a. Shows an extended lateral skin approach directly over the lesion. b. Demonstrates the tumor exposure by the elevation and the dorsal reflection of the fascio-muscular flap. c. Illustrates the location and the integrity of the posterior interosseous nerve. d. A fascial strip was warped around the radial shaft.

The surgical procedure was done under general anesthesia. The patient was put in a supine position. An extended lateral skin approach was utilized directly over the lesion. Subsequently, the anconeus muscle and the forearm extensors fascio-muscular flap were elevated sharply from the medial surface of the ulnar shaft and reflected volar to completely expose the bone lesion and the dislocated radio-humeral joint (Figure 3a, Figure 3b). The osteochondroma replaced the radial head and, with its broad base, extended to the radial neck and fused with the radius cortex. Throughout the dissection, the posterior interosseous nerve (PIN) was identified and protected (Figure 3c). The osteochondroma significantly distorted the radial head, the radial tuberosity, and the radial notch outside the ulna. Next, an osteotomy was done distal to the lesion to achieve a complete marginal resection of the tumor. The biceps tendon was reattached to the brachialis tendon. Lastly, a deep fascia strip from the right forearm was utilized to reconstruct a simulation of the annular ligament (Figure 3d). 

**Figure 4:**
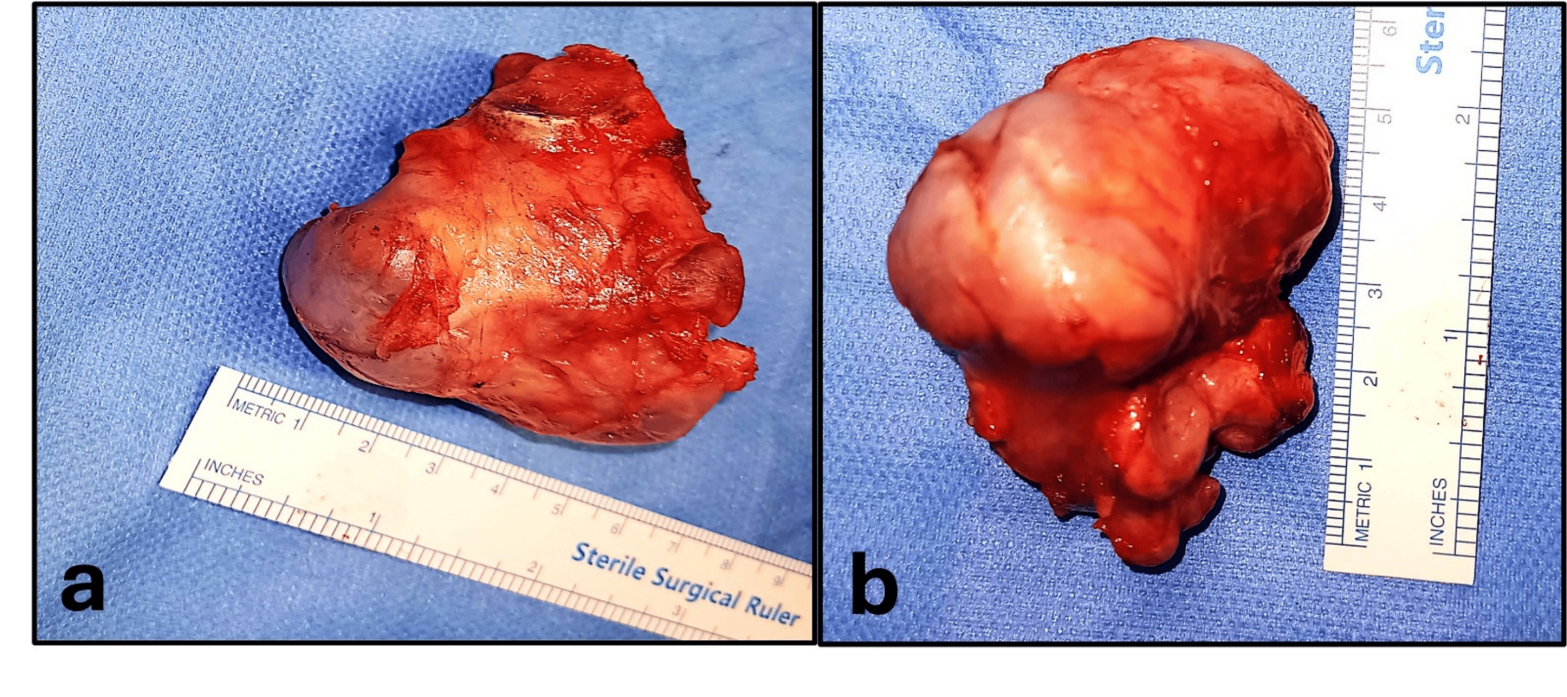
Size of the resected sample. Images a and b indicate the size of the resected sample, which is 7 cm wide and 6 cm long.

After the tumor resection, full flexion and extension of the elbow are carried out passively, and forearm rotation is moderately improved compared to the preoperative state due to abnormal articulation of the distal radioulnar joint. The histopathological examination of the resected specimen revealed osteochondroma, which was consistent with the preoperative diagnosis (Figure 4a, Figure 4b). 

**Figure 5:**
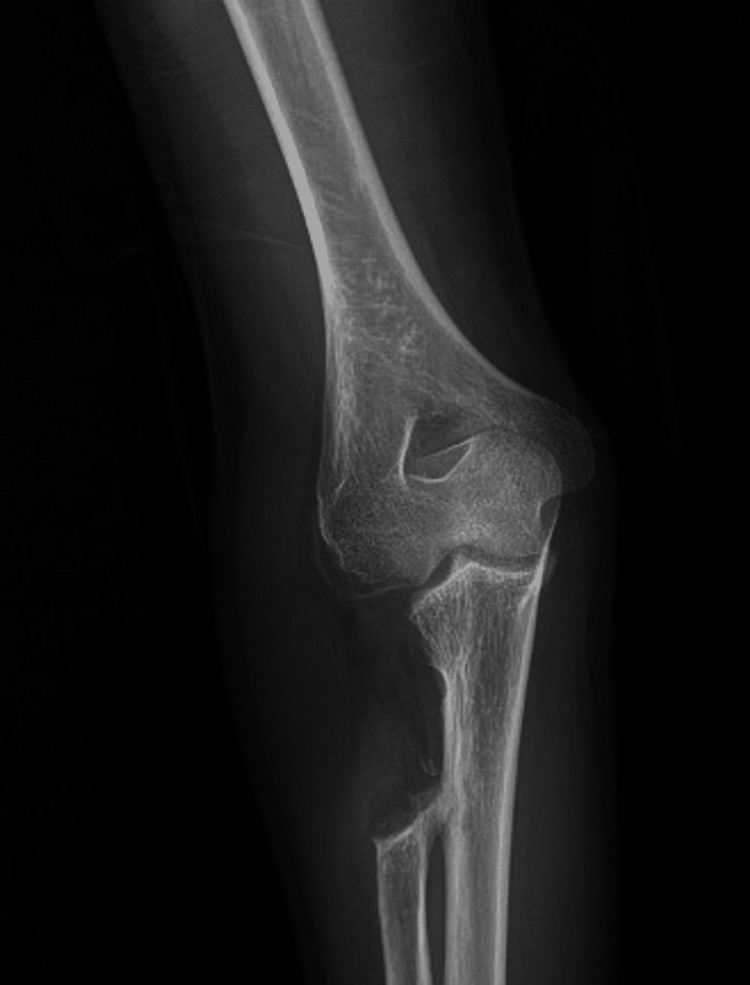
One-year postoperative plain radiograph. One-year postoperative plain radiograph of the right elbow joint showed mild elbow valgus deformity with no radial shaft proximal migration or tumor recurrence.

Postoperatively, a plaster cast was applied to the right upper limb, fixed with 90° of elbow flexion and forearm supination. Active functional exercises were started six weeks after surgery. During the follow-up, the plain radiograph showed mild elbow valgus deformity with no radial shaft proximal migration or tumor recurrence (Figure 5).

## Discussion

The severity of MHE does vary between individuals, and while no consensus has been reached regarding MHE classification, Mordenti et al. have devised a simple class system based on the deformities and functional limitations of sufferers to provide clarity between cases [7]. While class I has no deformities and no functional limitations, class II has deformities and no functional limitations. However, class III has both deformities and functional limitations. Moreover, each class is further subdivided into A and B, depending on severity.

MHE is a chronic condition characterized by persistent pain that significantly impacts both physical and emotional well-being. The pain associated with MHE disrupts daily activities, social interactions, and psychological health, leading to considerable disability. These challenges often result in a diminished quality of life, with the extent of functional limitations frequently underappreciated in clinical assessments [8].

To our knowledge, few cases of giant osteochondromas of the proximal radius have been reported in patients with MHE [9-11]. Table 1 summarizes the reported cases, highlighting their clinical presentations and management approaches. 

**Table 1:** Summarization of the reported cases of osteochondroma around the elbow joint.

Study	Study type	Tumor type	Age/gender	Main symptoms	Location of lesion	Shape/size	Procedure
Kim JP et al. [9]	Case report	Solitary osteochondroma	59/Female	Distal biceps tendon rupture	Lateral to radial tuberosity, left radius	Irregular, 2.0x1.0x0.5 cm	En bloc excision was performed, followed by anatomical reattachment of the distal biceps tendon to its insertion site using a bone anchor.
Niu XF et al. [10]	Case report	Solitary osteochondroma	12/Male	Chronic radial head dislocation	The inner rear side of radial neck, right radius	2.8x2.6x2.5 cm	Marginal tumor resection was performed. A rotational osteotomy with plate fixation was applied at the radial neck. A wedge osteotomy with Kirschner wire fixation addressed deformities in the radial shaft, followed by radial head reduction. Finally, annular ligament reconstruction was undertaken.
Oz O et al. [11]	Case report	Solitary osteochondroma	22/Male	Superficial radial neuropathy	Lateral aspect, right proximal radius	Not mentioned	Only analgesia and close follow up.
Current case	Case report	Part of MHE	15/Male	Swelling over the radial side, right elbow	Originating from radial head	Cauliflower-like, 5.1x4.1x4.8 cm	Excision of proximal radius with soft tissue reconstruction.

Radiologic predictors and key risk factors for radial head dislocation in MHE include isolated exostosis in the distal ulna or both the distal ulna and radius, radial or ulnar shortening >4.6 cm, radial bowing >8.1%, radial articular angle >35°, and the presence of ≥3 forearm exostoses [12]. We propose an additional risk factor: an exostosis originating from the radial head, which may further compromise stability and alignment. Identifying these predictors is essential for early intervention and tailored management strategies in patients with MHE.

There are no disease-modifying medical therapies for MHE. While asymptomatic osteochondromas typically require no intervention, surgery is necessary when they cause pain, neurovascular compression, functional limitations, or deformities [1]. Forearm symptomatic osteochondromas are often managed through surgical excision, ensuring clear margins by removing the exostosis, cartilage cap, and perichondrium, effectively minimizing recurrence [4].

Osteochondroma of the proximal radius causing radial head dislocation can be managed through radial head excision, corrective osteotomy at two levels, and annular ligament reconstruction using the palmaris longus tendon. In the reported case, persistent proximal radioulnar synostosis necessitated arthroplasty of the proximal radioulnar joint using a silicone sheet one year later. While this approach yielded satisfactory cosmetic results, forearm rotation remained severely restricted [13].

External fixation is a proven method for managing MHE-related forearm deformities. Techniques like unilateral external fixation with osteochondroma resection and ulnar lengthening were prescribed to improve function and radiological outcomes [14]. Modified ulnar lengthening with Illizarov fixators effectively addresses Masada type 2 deformities, achieving radial head relocation, joint realignment, and spontaneous forearm correction [15]. The Illizarov frame, using two rings for controlled ulnar osteotomy and lengthening without tumor excision, enabled spontaneous radial head reduction and forearm deformity correction [16].

Additionally, distraction osteogenesis is an effective technique for managing forearm deformities in MHE. Studies highlight its utility in ulnar lengthening combined with angular correction of the radius and ulna to improve bowing, correct carpal slip, and restore elbow alignment [17,18].

Recently, three-dimensional (3D) printing has been an innovative tool in surgical planning, offering precise anatomical models that illustrate lesions, tumor features, and critical structures. By enhancing preoperative visualization and understanding, it aids in complex decision-making, though further research is needed to validate its clinical effectiveness [19].

Furthermore, simplified approaches without radius osteotomy also show significant functional and anatomical improvements, especially early post-dislocation [20]. Wide local excision of the proximal radius, including the radial head, has been described in the management of a case of a chondromyxoid fibroma rather than an osteochondroma. This approach successfully restored painless functional movement and allowed the patient to resume independent daily activities [21].

In our case, we applied this technique to restore the elbow range of motion and enhance forearm rotation, successfully enabling the patient to achieve functional supination and pronation.

After one year of follow-up, our patient exhibited no clinical or radiographic signs of proximal radial migration, emphasizing the stability achieved. The central band of the interosseous membrane, responsible for 71% of longitudinal stiffness after radial head excision, likely played a critical role in maintaining the alignment [22]. In addition, the presence of distal radioulnar joint synostosis as part of MHE could play a role in the stability and prevent proximal radial migration.

A previous study on patients undergoing primary radial head resection with long-term follow-up (15 years) reported wrist pain and radial migration in only three cases [23]. These findings reinforce the need to protect soft tissue attachments to ensure joint stability and minimize postoperative complications.

## Conclusions

This case highlights the successful surgical management of a giant osteochondroma of the proximal radius in a patient with multiple hereditary exostoses (MHE). Surgical excision of the tumor effectively restored joint mobility and improved forearm function. A one-year follow-up revealed no recurrence or proximal radial migration. This case provides valuable insights into the limited literature on proximal radius osteochondromas, emphasizing the importance of ongoing research and advancements in surgical techniques to optimize outcomes in similarly challenging cases.
